# The physiological response during optogenetic-based cardiac pacing in awake freely moving mice

**DOI:** 10.3389/fphys.2023.1130956

**Published:** 2023-09-06

**Authors:** Jun Kaminosono, Yuki Kambe, Akihide Tanimoto, Tomoyuki Kuwaki, Akira Yamashita

**Affiliations:** ^1^ Department of Physiology, Kagoshima University Graduate School of Medical and Dental Sciences, Kagoshima, Japan; ^2^ Department of Pharmacology, Kagoshima University Graduate School of Medical and Dental Sciences, Kagoshima, Japan; ^3^ Department of Pathology, Kagoshima University Graduate School of Medical and Dental Sciences, Kagoshima, Japan; ^4^ Department of Medical Neuropharmacology, Wakayama Medical University School of Pharmaceutical Sciences, Wakayama, Japan

**Keywords:** optogenetics, cardiac pacing, pacemaker, heart rate, respiration, blood pressure

## Abstract

There are several methods to control a heart rate, such as electrical stimulation and drug administration. However, these methods may be invasive or affect other organs. Recently, an optogenetic-based cardiac pacing method has enabled us to stimulate the cardiac muscle in non-contact. In many previous studies, the pacing was applied *ex vivo* or in anesthetized animals. Therefore, the physiologic response of animals during optogenetic pacing remains unclear. Here, we established a method of optogenetic-based cardiac pacing in awake, freely moving mice and simultaneously measured electrocardiogram, blood pressure, and respiration. As a result, light-induced myocardial contraction produces blood flow and indirectly affects the respiration rhythm. Additionally, light illumination enabled heart rate recovery in bradycardic mice. These findings may be employed for further research that relates a heartbeat state to animal behavior. Together, this method may drive the development of less invasive pacemakers without pacing leads.

## Introduction

Optogenetics has been widely employed as a tool to control cellular function (Boyle, Karathanos, and Trayanova, 2018) by a photostimulation of the light-gated ion channel (e.g., channelrhodopsin 2 (ChR2)) expressed in specific cells induces ion influx (Nagel et al., 2003). This is mainly applied to the neural system (Boyden et al., 2005; Li et al., 2005; Nagel et al., 2005), the skeletal muscles (Bruegmann et al., 2015) and the cardiac muscles (Bruegmann et al., 2010). ChR2 can depolarize cardiomyocytes in the illuminated area and induce heartbeats via gap junctions (Bruegmann et al., 2010). Therefore, using optogenetics, we can control the heartbeat at a desired timing, like an artificial pacemaker.

Applying optogenetics to the cardiac pacemaker has several advantages. First, we can stimulate the cardiac muscle without pacing leads. The surgery for attaching pacing leads to an animal’s heart is difficult and invasive because it needs thoracotomy. Second, we can stimulate specific cells. In humans, electric current from pacing leads sometimes leaks to other tissues, causing complications such as convulsions (Bayliss et al., 1968; Tanabe et al., 2019; Dalex et al., 2021). Drugs that alter heart rate may affect non-specific organs and cause side effects. Applying optogenetics, we can stimulate only ChR2-expressing tissue with blue light; in this case, the cardiac muscle. For these reasons, a leadless and non-electrical pacemaker may be useful.

Optogenetic-based cardiac pacing has been widely studied as a novel, minimally invasive technology, mainly for treating cardiovascular diseases (e.g., the light intensity required for pacing, the photostimulation’s duty cycle, and how the stimuli spread throughout the heart (Bruegmann et al., 2010; Nussinovitch and Gepstein, 2015; Vogt et al., 2015; Zaglia et al., 2015; Gutruf et al., 2019; Li et al., 2021)). In these studies, cardiac pacing has mainly been applied to cultured cardiac tissue, the Langendorff perfused heart, and anesthetized mice and rats. However, there are not many studies assessing the animals’ physiological response during optogenetic-based pacing (Hsueh et al., 2023), as optogenetic-based cardiac pacemakers have rarely been applied to conscious, freely moving animals.

In this study, we established a method for optogenetic-based cardiac pacing in freely moving ChR2-expressed mice and examined the mice’s physiological response (heart rate, respiration, and blood pressure) during pacing. Furthermore, we verified and discussed whether this pacing system works as an artificial pacemaker. During our study, Hsueh et al. reported the method of optical pacing using red-light sensitive opsin (Hsueh et al., 2023). We hope to contribute to the field by adding our findings.

## Materials and methods

### Animals

All experimental procedures were performed under the guiding principles for the care and use of animals in the field of physiological sciences published by The Physiological Society of Japan and were approved by the Institutional Animal Use Committee at Kagoshima University (MD18011, MD20004).

Male and female wild-type mice (C57BL/6) were used for this study. Forty-seven mice were divided into the channelrhodopsin 2-expressing (ChR2(+)) group (*n* = 22) and ChR2(−) (non-injected control) group (*n* = 18). Three-day-old mice of ChR2(+) group were injected with adeno-associated virus (AAV), as described below. Device implantation and photostimulation were performed from 8 weeks to 1 year after the injection of the AAVs. The animals were maintained in the laboratory under standard conditions: 12/12-h cycle (lights on at 7:00 a.m. and off at 7:00 p.m.), the temperature at 24°C ± 1°C, and food and water *ad libitum*. Efforts were made to minimize animal suffering and the number of animals used.

### AAV vector

AAVs expressing Cre-recombinase depending on cardiac troponin T (cTnT) promoter activity and expressing ChR2/mCherry depending on Cre-recombinase were used in these experiments. AAV-FLEX-rev-ChR2(H134R)-mCherry was a gift from Scott Sternson (Addgene plasmid #18916; http://n2t.net/addgene:18916; RRID:Addgene_18916) (Atasoy et al., 2008), and pAAV.cTNT.iCre was a gift from William Pu (Addgene plasmid #69916; http://n2t.net/addgene:69916; RRID:Addgene_69916) (Lin et al., 2015). These AAV vectors (serotype 9) were produced using the AAVpro^®^ Helper Free System (Takara Bio Inc., Shiga, Japan). After mixing the same amount of plasmid DNA (pAAV, pRC9, and pHelper), the mixture of plasmids was transiently transfected into AAVpro^®^ 293T Cells (Takara Bio Inc., Shiga, Japan) using 1 mg/mL polyethyleneimine (Polyscience, Eppelheim, Germany) according to the manufacturer’s instructions. Three days after transfection, the 293T cells were harvested, washed, and lysed in phosphate buffered saline (PBS) by freeze/thaw cycles. Cell lysates were treated with benzonase (Merck Millipore, Darmstadt, Germany) to degrade contaminated DNA and RNA. Subsequently, Opti-prep (Abbott Diagnostics Technologies AS, Oslo, Norway) gradient ultracentrifugation was used to purify AAV virions. The AAV virions were then concentrated using Vivaspin^®^ 20 (Sartorius, Tokyo, Japan), and AAV titers were quantified as previously described (Zincarelli et al., 2008). AAV vectors were diluted with Hanks’ balanced salt solution to a concentration of 2.0 × 10^13^ GC/mL. The samples were then aliquoted and maintained at −80°C until further use.

Prior to injection, the AAV vectors were placed on ice for 30 min. Subsequently, AAVs (50 μL of AAV-cTnT-Cre and 50 μL of AAV-CAG-FLEX-ChR2-mCherry, totaling 100 μL) were intraperitoneally injected into 3-day-old mice with a 30-G needle.

### Preparation of the LED device

#### The LED device construction

**FIGURE 1:**
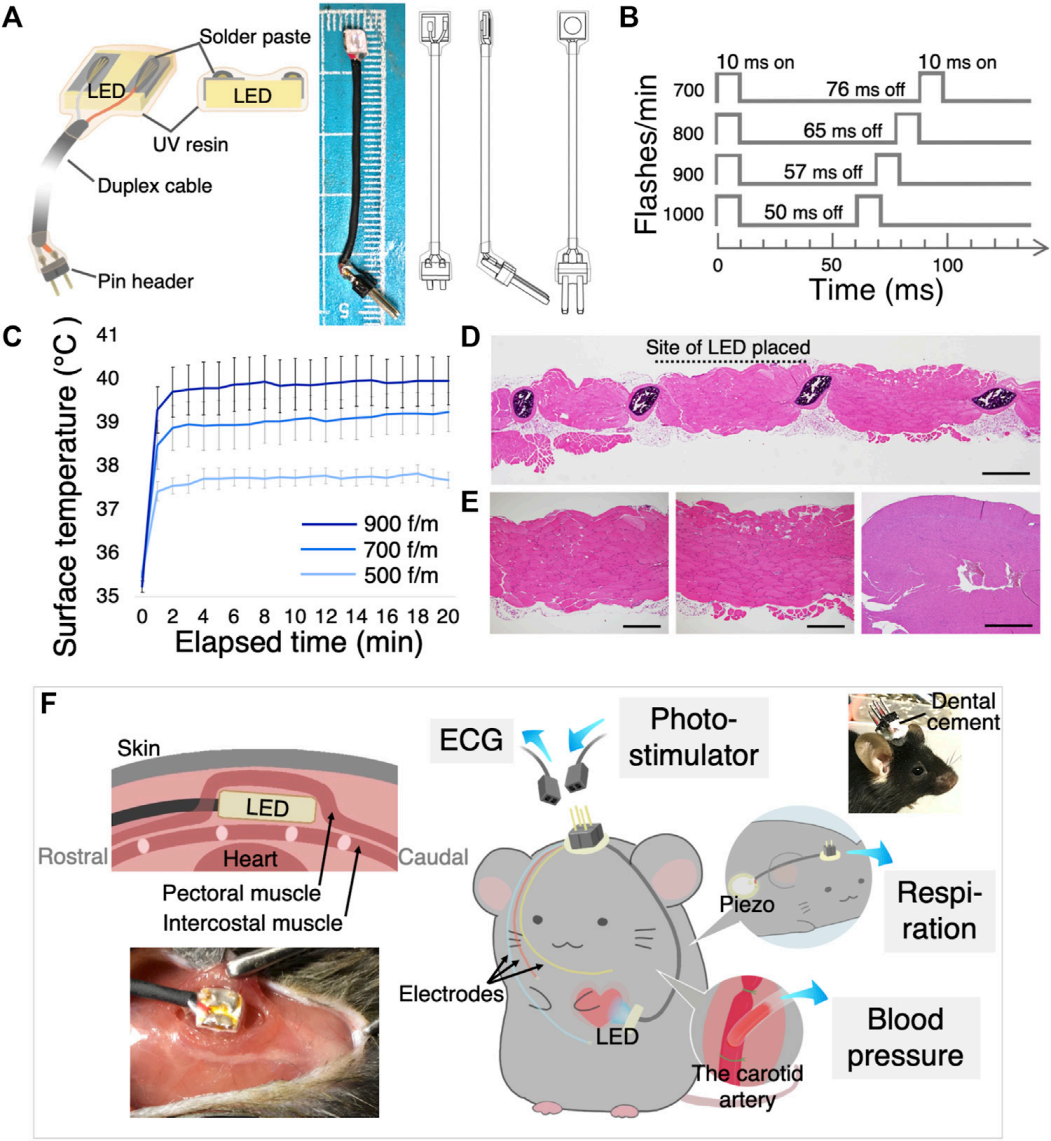
Design of the implantable LED device and surgical procedure. **(A)** The illustration shows the main components of the LED device (left), an image and illustrations of the LED device (right). **(B)** Various optogenetic stimulation frequencies and duty cycles. **(C)** LED surface temperature measurement. All flash durations were set at 10 ms, and flashing rates were set at 500, 700, and 900 flashes/min (f/m). Data are shown as the mean ± SEM (*n* = 5). **(D)** Histological examination of intercostal muscle and heart when the LED was illuminated *in vivo* for 30 min at 900 f/m. Scanning view of the intercostal muscle. The site of probe placement is shown by the dotted line. Scale bar: 1 mm. **(E)** Higher power views of the striated muscles at the site of the LED (left) and at the site of no LED (middle), showing no pathological change. Scale bar: 500 μm. Right) The histological section of the heart had no necrosis or inflammation. Scale bar: 2 mm. **(F)** Schematic illustration and photograph shows how the LED device, ECG electrodes, the piezo sensor, and the vascular cannula were implanted. Sockets were connected to the computer. LED, light-emitting diode; ECG, electrocardiogram; SEM, standard error of the mean.

A monochromic high-power (37 lm/W) and blue-light (475 nm) light-emitting diode (LED) was selected for stimulation (LUXEON 3535L, LUMILEDS, San Jose, United States). The LED was soldered to a duplex cable (AWG36, Mogami Wire & Cable Corp, Nagano, Japan) and coated with clear UV resin (Daiso, Hiroshima, Japan) to prevent electric leakage and immersion in the tissue fluid (Figure 1A). This coating should be as thin and flat as possible for stable LED placement on the intercostal muscles. These cables were soldered to pin headers (Switchscience, Tokyo, Japan), and the soldering sites were coated with a clear UV resin. A multimeter (M320, Mastech, United States) was used to ensure that the LED was insulated by the resin coating. The wire for monitoring the electrocardiogram (ECG) (AWG32, Mogami Wire & Cable Corp, Nagano, Japan) was also soldered to the pin header. This tip of the wire was slightly exposed. Additionally, a microcomputer (Arduino UNO, Arduino, Italy) was used as the pulse generator. The LED driver (PlexonBright LD-1, Plexon, Texas, United States) supplied a 200 mA electric current to the LED.

#### The light intensity measurement

The light intensity was measured using an optical power meter (PM20A, Thorlabs, Tokyo, Japan). In experiments to measure the capture rate with light intensity, photostimulation was performed over the intercostal muscles under anesthesia to keep the constant distance between the LED and the heart.

#### The LED surface temperature measurement

The surface temperature of the LED was measured using a thermometer (BWT-100, Bio research center, Nagoya, Japan). For animal welfare reasons, this experiment was performed using agar gel. Placed a 1 mm thick and 2 cm^2^ agar gel (Kitahara Industry, Okayama, Japan) on a 39°C hot plate (MHP-45, Masuda, Osaka, Japan), the gel surface temperature was 35°C. An ultra-thin thermometer (IT-18, Physitemp, North America) was placed on the gel, and the LED was placed directly above the thermometer. They were then covered with a 1-mm thick gel from above. The LEDs were blinked for 20 min at three different rates.

### ECG and LED device implantation

Eight weeks after AAV injection, mice were anesthetized (1.5% isoflurane, inhalation) for LED-device implantation. The skin and pectoral muscles were slightly incised, and the LED device was positioned over the intercostal muscle above the left ventricle (LV) using a bio-adhesive (Vetbond 3M, Tokyo, Japan).

The ECG electrodes were positioned where signals could be obtained. The ECG measurement cables were sutured to the pectoral muscle. The cables and LED devices were placed subcutaneously, extending from the chest to the back of the neck. The cable terminals were connected with pin-headers and placed above the skull and fixed using dental cement (Unifast Trad, GC Japan, Tokyo, Japan). For clearer ECG measurements, two mice had telemeters (TA11ETA-F10, Data Sciences International, MN, United States) implanted in abdominal cavities a day after the LED device implantation.

After all surgeries, the mice were administered an antibiotic (penicillin G, 40,000 U/kg body weight, Nacalai Tesque, Kyoto, Japan) and analgesic (buprenorphine, 0.04 μg/kg body weight, Otsuka Pharmaceutical, Tokyo, Japan).

### Optogenetic-based cardiac pacing

One week after the device implantation, photostimulation was performed for freely moving, conscious mice with blue light at various frequencies. Pin headers from the LED device were connected to a pulse generator, and the ECG electrodes were connected to an oscilloscope (VC-11, Nihon Kohden, Tokyo, Japan).

### Respiration and blood pressure measurement

According to our previously published method, respiration (Ikoma et al., 2018) and blood pressure (Zhang et al., 2006) were measured in the mice implanted with the device. Methods of implanting devices to measure respiration and blood pressure are described below. The mice were anesthetized with 1.5% isoflurane for the surgery.

To measure the respiratory movements, a piezo sensor (7BB-12-9, Murata Manufacturing Co., Ltd. Tokyo, Japan) with duplex cables was implanted into the dorsal thorax of mice. Output wires from the piezo sensor were connected to connector pins. The connector was secured to the skull using dental cement (Unifast Trad, GC Japan, Tokyo, Japan). One week after the device implantation, photostimulation was performed for freely moving mice.

To measure blood pressure, the catheter was inserted into the carotid artery. The details are as follows. An incision was made in the skin of the neck. The vagus nerve, jugular vein, and other structures were carefully separated from the artery, and the right carotid artery was exposed. The tip of the catheter (sp31 polyethylene tube, Natsume, Tokyo, Japan), which was filled with heparinized saline, was inserted into the carotid artery, and the insertion site was tightly ligated. The opposite end of the catheter was placed subcutaneously into the back of the neck and attached to the skin. The catheter was connected to a pressure transducer (AP-620G; Nihon-Kohden, Tokyo, Japan) to record pulsatile blood pressure. Photostimulation was performed after the mice awoke from anesthesia. To induce bradycardia while measuring blood pressure, muscarine (0.05 mg/kg body weight) was intraperitoneally administered.

Only site-specific optical pacing during blood pressure measurement (Supplementary Figure S4) was performed under thoracotomy. Mice were artificially ventilated (Minivent type 845, Hugo Sachs Electronik, Germany) using a tracheal cannula. Following a thoracotomy, the LED was placed approximately 2 mm above the left ventricle. By comparing the Area under the Curve (AUC) of the blood pressure chart per unit time while pacing with the AUC immediately before pacing, the relative change in the blood pressure was calculated.

### Histology

The mice were deeply anesthetized with urethane (2.0 g/kg, i. p.) and perfused via the postcaval vein with 20 mL of 0.01 M PBS containing 20 unit/mL heparin followed by 20 mL of chilled 4% paraformaldehyde in 0.01 M PBS (pH 7.4). The hearts were then removed and post-fixed at 4°C overnight. The hearts were sliced into sections of 40-µm thickness using a vibratome (D.S.K. Kyoto, Japan) and collected in PBS. Floating immunohistochemical staining was performed as follows: sections were sequentially incubated in PBS containing 0.3% Triton-X and 1% normal donkey serum for 30 min, rabbit anti cardiac troponin T antiserum (1/200, 26592-1-AP, Proteintech, Japan) overnight at 4°C, and CF488-conjugated anti-rabbit IgG (1/200, Biotium, United States) for 2 h at room temperature in a dark box. After incubation, the sections were washed and mounted onto glass slides. We observed the sections with a fluorescence microscope (LSM 700, ZEISS, Tokyo, Japan, BZ-X710, Keyence, Osaka, Japan). The expression rate of ChR2 was measured by calculating the ratio of the mCherry-expressed area to the cTnT-expressed area using ImageJ (Wayne Rasband, Washington, D.C., United States).

At necropsy to check for inflammation, the chest wall where the LED was placed and the heart were resected immediately after illuminating LED for 30 min at 900 flashes per minute (f/m) in anesthetized mice. Then, the tissues were fixed with 10% neutral phosphate-buffered formalin, routinely processed for paraffin embedding, and sectioned and stained with hematoxylin and eosin (HE). To examine inflammation in more detail, floating immunohistochemical staining was performed as described above. The chest wall and the heart were resected 2 days after illuminating LED for 30 min at 900 f/m. Sections were sequentially incubated in PBS containing 0.3% Triton-X and 1% normal donkey serum for 30 min, rabbit anti F4/80 antiserum (1/200, 28463-1-AP, Proteintech, Japan) and rat anti GR1 antiserum (1/200, 65140-1-lg, Proteintech, Japan) overnight at room temperature, and CF568-conjugated anti-rabbit IgG (1/200, Biotium, United States) and Alexa 488-conjugated anti-rat IgG (1/200, Invitrogen, United States) for 2 h at room temperature.

### Statistical analysis

The ECG signals went through high- (1 kHz) and low-cut (1.5 Hz) filters, and were amplified by an amplifier. Respiration signals from the piezo sensor went through a high-cut (10 Hz) filter. The acquired signals were digitized by an A/D converter (Mac Lab, ADInstruments Inc., Bella Vista, NSW, Australia) at 1 kHz and recorded on a computer with LabChart version 7 software (AD instruments). In HR graphs, smoothing window width was set at 501 samples. Statistical comparisons were performed using a two-tailed *t*-test or one- or two-way ANOVA with *post hoc* Bonferroni’s multiple comparison tests or repeated measures ANOVA with *post hoc* Tukey’s multiple comparison tests using Prism 5 software (GraphPad Software, Inc.). *p* < 0.05 was considered significant.

## Results

### Development of implantable devices for freely moving mice

To illuminate a blue light on the cardiomyocytes in freely moving mice, we developed an implantable pacing device. The device incorporates a small, thin, and high-powered LED chip. The detailed scheme in Figure 1A shows that the LED chip surface was coated with a clear UV resin to prevent electrical leakage and deterioration of the LED. The light intensity of supplying power to the LED was set to approximately 40 mW/mm^2^ to deliver to the cardiomyocyte enough light. The light intensity on the cardiomyocyte through the intercostal muscles was approximately 1.5 mW/mm^2^. A higher light intensity was more effective in synchronizing the heartbeats with light-pulse (Supplementary Figure S3A), as a previous study reported (Nussinovitch and Gepstein, 2015).

As the high-intensity LED produces heat well, we measured the surface temperature over time with a 10 ms flash duration (Figure 1B). Figure 1C shows the surface temperature of the blinking LED for 20 min continuously at three different frequencies. The temperature rose rapidly over the first 2 min and nearly plateaued. Additionally, even with blinking at high frequencies, the surface temperature did not exceed 40° (Figure 1C). These LED heat generation induce no significant changes in the myocardial tissue (Figure 1E; Supplementary Figure S1A). The surrounding sections after illuminated revealed normal histology with no necrosis or inflammation of the intercostal muscles that were in contact with the LED, as well as in the adjacent tissues without LED contact (Figures 1D, E; Supplementary Figure S1A).

Consequently, we considered that this original LED device could be used in freely moving mouse experiments describing below. Figure 1F shows the implantation method.

### Verification of ChR2 expression in mice by AAV systemic administration

**FIGURE 2:**
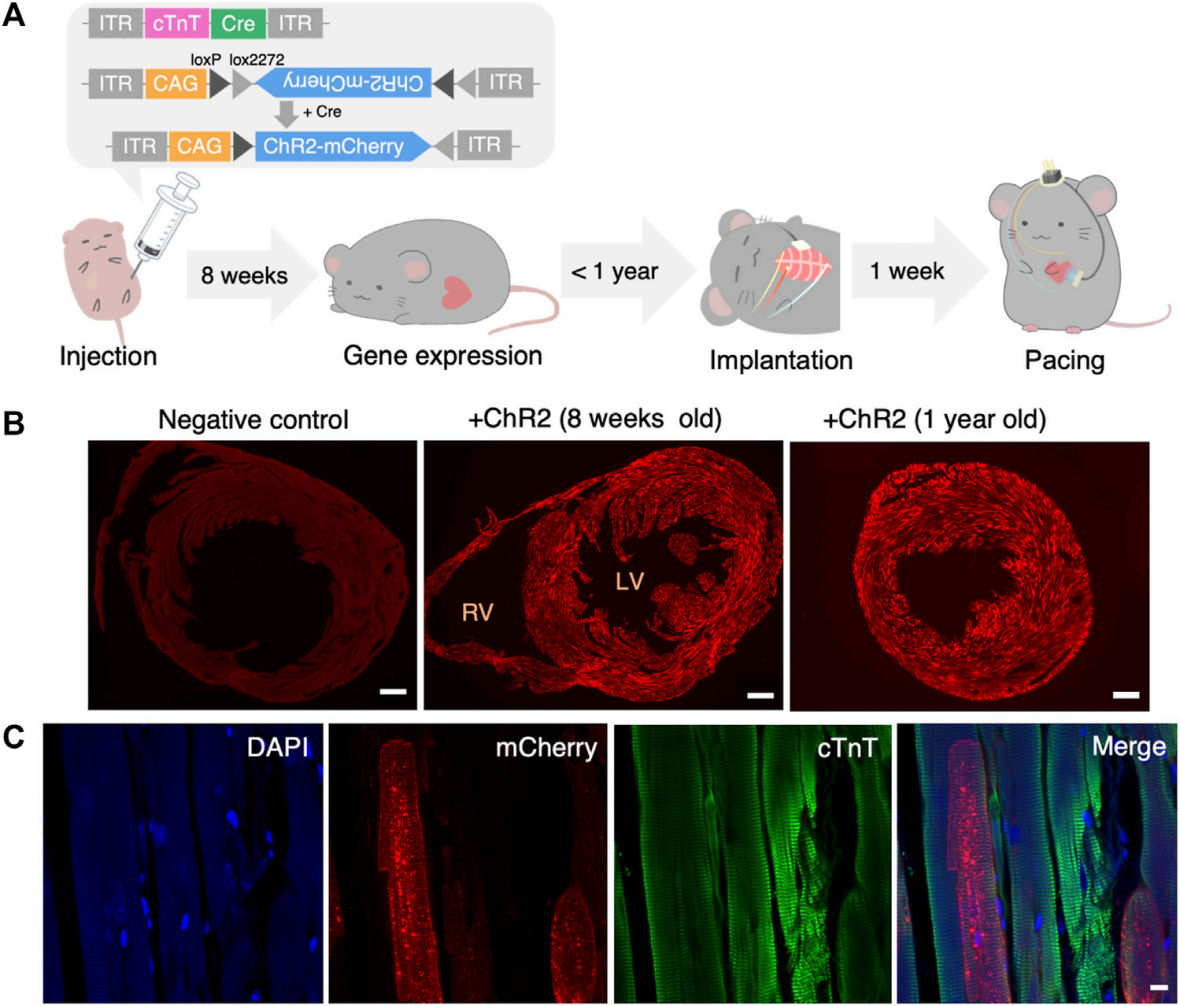
Systemic administration of AAV and confirmation of gene expression. **(A)** Structure of administered AAV and the timeline of the experiment. **(B)** Representative fluorescence images of the heart slice sample of negative (non-injected) control (left), 8 weeks (middle) and 1 year (right) after systemic AAV administration. Red: mCherry. Scale bar: 500 μm. **(C)** Representative images showing mCherry (red), anti-cTnT staining (green), DAPI (blue) and a merged image of the heart 1 year after systemic AAV administration. Scale bar: 10 μm. AAV, adeno-associated virus; LV, left ventricle; RV, right ventricle; cTnT, cardiac troponin T; DAPI, 4′,6-diamidino-2-phenylindole.

An AAV encoding cTnT-dependent Cre-recombinase and an AAV encoding Cre-dependent ChR2/mCherry were used in this experiment to induce cardiomyocyte-restricted transgene expression (Figure 2A). Eight weeks after the injection in 3-day-old mice, we confirmed fluorescence expression in all mice. Figure 2B shows the widespread transduction of ChR2/mCherry to cardiomyocytes 8 weeks or 1 year after AAV administration. The expression rate of ChR2/mCherry after 1 year was 53.7% ± 5.74% (9 samples in 5 mice). More detailed images of cardiac sections are shown in Supplementary Figures S1B, C. Figure 2C indicates the specific expression of ChR2 in cardiomyocytes. Next, we checked the potential off-target effects of these AAVs in several organs systemically. We found that the AAVs expressed fluorescence in both the cardiac and the skeletal muscles (55.1% ± 4.73% (5 samples in 5 mice)), but not in the digestive tract or brain (Supplementary Figure S2).

### Optogenetic cardiac control in freely moving mice

**FIGURE 3:**
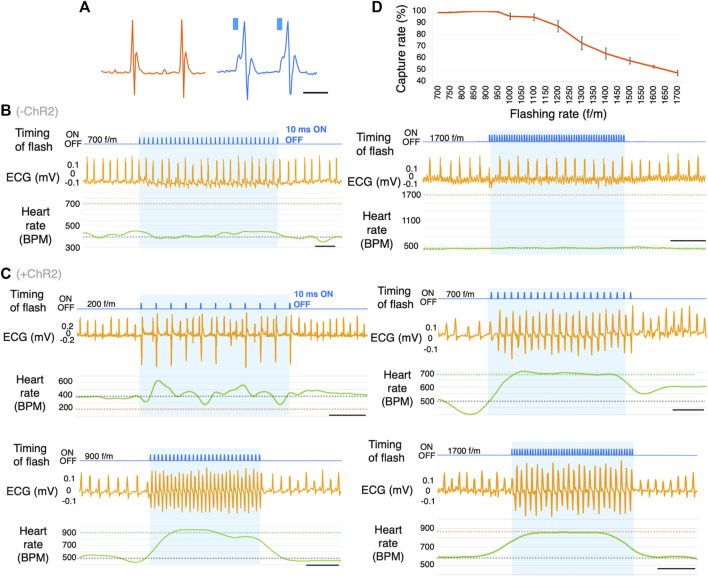
Optogenetic-based cardiac pacing in freely moving mice. **(A)** Comparison of natural waveforms and light-induced waveforms of ECG. These waves were measured using a telemetry system. Blue columns represent the timing of light illumination. **(B)** Representative ECG traces in response to photostimulation at the frequency of 700 and 1700 flashes/min (f/m) in ChR2(−) mice. The blue shading indicates the duration of photostimulation. The gray dotted line on the BPM chart indicates the baseline heart rate. The orange dotted lines indicate the frequency of photostimulation (*n* = 16). scale bar: 0.5 s. **(C)** Representative ECG traces in response to photostimulation at the frequency of 200, 700, 900, and 1700 f/m in mice expressing ChR2. The blue shading indicates the duration of photostimulation. The gray dotted line on the BPM chart indicates the baseline heart rate. The green and orange dotted lines indicate the frequency of photostimulation (*n* = 19). scale bar: 0.5 s. **(D)** Dose-response curve summarizing the effects of flashing rate. Data are presented as the mean ± SEM (total 26-27 trials in 7 mice per f/m). ECG, electrocardiogram; BPM, beats per minute.

The heartbeat was synchronized with light pulse stimulation (each stimulation period was <5 s, the processes shown in Figure 1B) provided by the implanted LED device in freely moving mice (*n* = 19, Supplementary Movie S1). All ChR2(+) mice used in this study could repeatedly do photo-pacing (Supplementary Figure S3B). The mice were illuminated several times at different frequencies. Figure 3A shows the spontaneous ECG traces and the ECG during photostimulation. A slightly wider QRS complex without a P wave was observed following the stimulation artifact. In ChR2(−) mice, photostimulation did not change the heart rate (Figure 3B). In ChR2-expressed (ChR2(+)) mice, the photostimulation and heartbeat were almost completely synchronized in 700–1,200 f/m stimulation frequency (Figure 3C). Stimulation at a lower frequency than baseline (about 400–600 beats per minute (BPM)) did not slow down the heartbeat. Additionally, stimulation at a higher frequency (>1,400 f/m) induced heartbeats once every two pulses. Figure 3D shows the capture rate (percentage of light-induced R waves divided by the number of flashing lights). The capture rate decreased as the flashing rate increased (*n* = 7). While pacing, no abnormal behavior was observed in these mice. Nor did any long-term behavior problems were observed.

### Respiration measurement and the effects of photostimulation on the pectoral muscles

**FIGURE 4:**
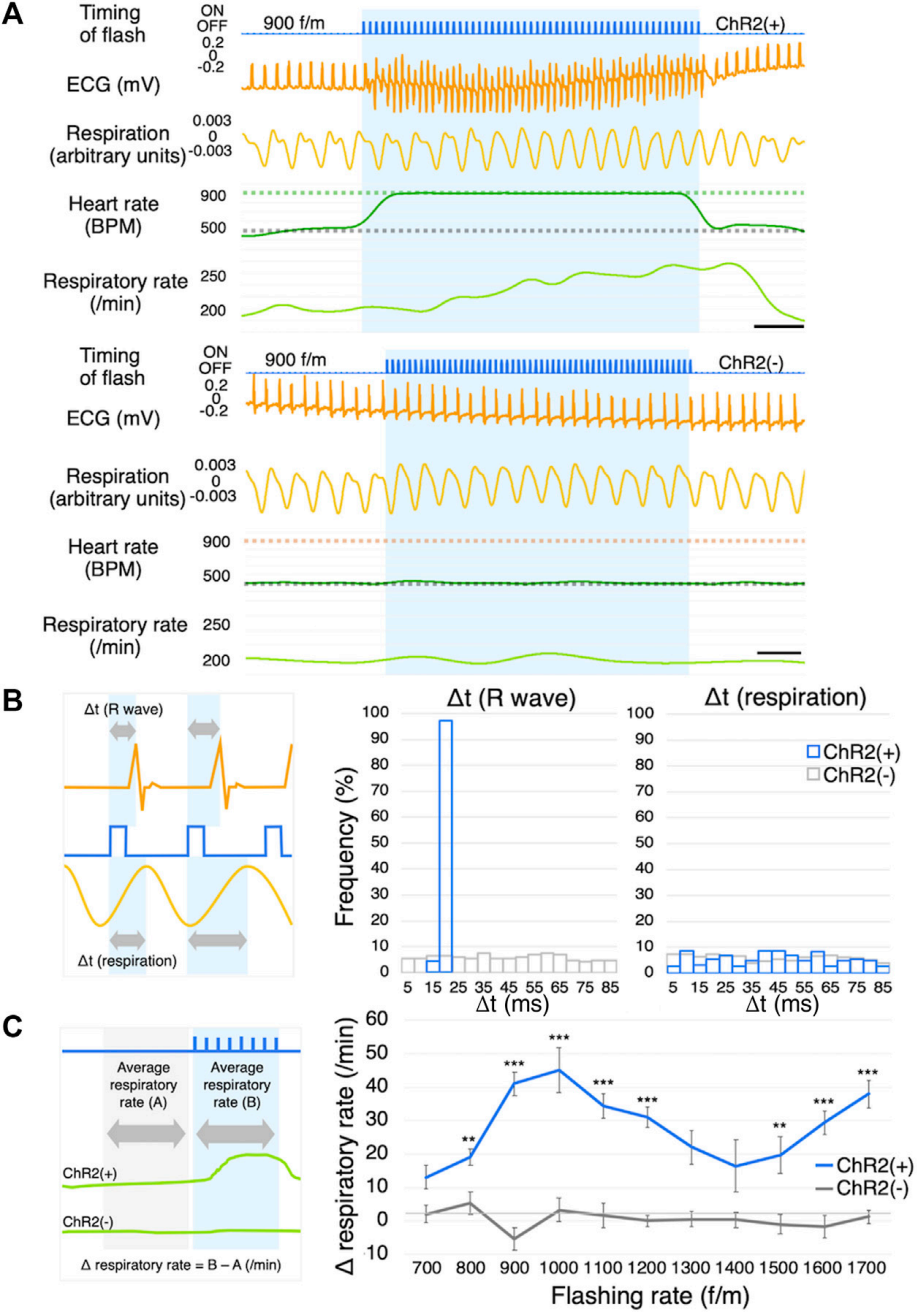
Respiration measurement. **(A)** Representative ECG and respiration traces of ChR2(+) mice (upper) and ChR2(−) mice (lower) in response to photostimulation at the frequency of 900 f/m. The blue shading indicates the duration of photostimulation. The gray dotted line on the BPM chart indicates the baseline heart rate. The green and orange dotted lines indicate the frequency of photostimulation. ChR2(+); *n* = 5, ChR2(−); *n* = 4, scale bar: 0.5 s. **(B)** Explanation of Δt (left), histograms of Δt of R waves (middle) and respiration (right). Gray arrows indicate the length from the start of light illumination until the peak of the R wave and breathing. Orange line: ECG; Blue line: timing of flash; Yellow line: respiration. **(C)** Explanation of Δ respiratory rate (left), Dose-response curve summarizing the effects of flashing rate (right, ChR2(+); *n* = 5, ChR2(−); *n* = 4, 10-46 trials per f/m in 9 mice). Data are presented as the mean ± SEM. Two-way ANOVA indicated that there was a significant statistical difference among regions (F (10,404) = 3.323, *p* < 0.001). Bonferroni’s multiple comparison test was used as a *post hoc* test (**p < 0.01, ***p < 0.001). ECG, electrocardiogram; BPM, beats per minute; ANOVA, analysis of variance.

As mentioned above, ChR2 was expressed in the skeletal muscles. Since the LED was positioned on the pectoral muscle, we next explored whether photostimulation affects respiration (ChR2(+); *n* = 5, ChR2(−); *n* = 4) (Figure 4A). We show histograms of the duration from the timing of a light flash to the next R wave and instance of breathing (Δt) (Figure 4B). As a control group, ChR2(−) mice were photostimulated using the same surgery. The flashing rate was set at 700 f/m. If the photostimulation directly affected respiration by moving the pectoral or the breathing-related muscles, the histogram’s distribution would have been different between the ChR2(+) and ChR2(−) groups. In the respiration histogram, there was no difference between the two groups (Figure 4B, right). Conversely, the R wave histogram had one peak at 15–20 ms in the pacing group (Figure 4B, middle). R waves appeared with a delay of 18 ± 0.14 ms from the onset of the flash. We measured the respiratory rate while pacing at 700–1700 f/m, and calculated the value (Δ respiratory rate) obtained by subtracting the average respiratory rate immediately before photostimulation from that during photostimulation (Figure 4C, left). Averages before stimulation at all flashing rates of 700–1700 f/m (i.e., the average of “A” in Figure 4C) were 180 ± 5.19 BPM in the ChR2(+) mice and 207 ± 8.19 BPM in the ChR2(−) mice. The result showed that the respiratory rate increased significantly at all flashing rates in the ChR2(+) mice (Figure 4C, right). Notably, the respiratory rate did not increase in linear to the flashing rate. More detailed comparison data for ChR2(+) and ChR2(−) mice are shown in Supplementary Figure S5.

### Blood pressure measurement and bradycardia treatment

**FIGURE 5:**
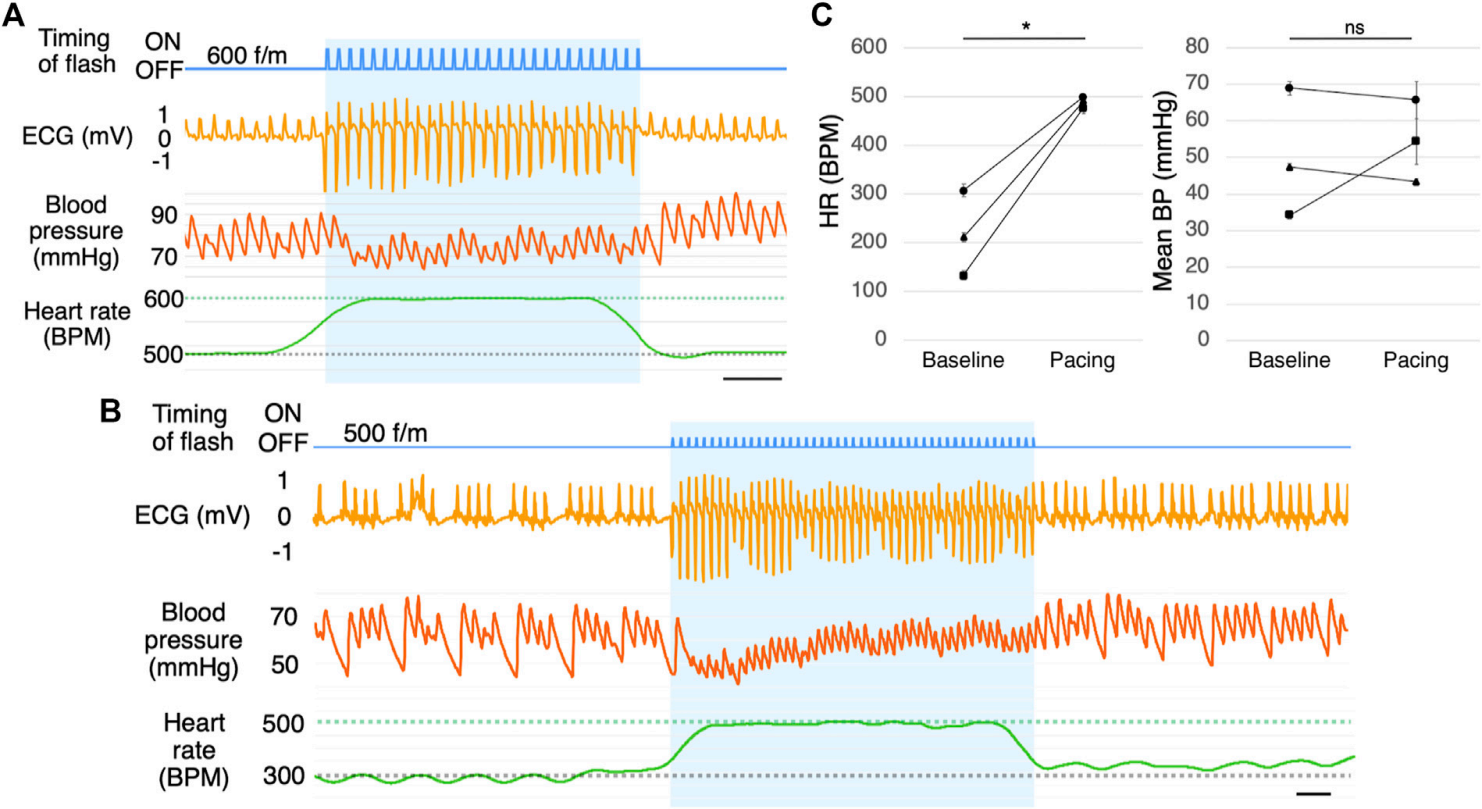
Blood pressure measurement. **(A)** Blood pressure measurement during photostimulation (*n* = 5). Scale bar: 0.5 s. **(B)** Representative ECG of a mouse with muscarine-induced bradycardia with photostimulation at 500 f/m (*n* = 3). Scale bar: 0.5 s. **(C)** Mean heart rate (left) and mean blood pressure (right) with photostimulation at 500 f/m under muscarine-induced bradycardia. Baseline refers to the time immediately before photostimulation and pacing refers to the time during photostimulation. Each dot represents the average of four samples from one mouse. Data are presented as the mean ± SEM (*n* = 3, *p < 0.05, paired *t*-test). ECG, electrocardiogram; RA, right atrium; RV, right ventricle; LV, left ventricle; BP, blood pressure; SEM, standard error of the mean.

Stimulation at a frequency beyond the biological range may cause a pulse deficit. It is possible that blood is not being pumped and the pulse is absent occasionally, although the ECG appears normal. We, therefore, verified whether the blood is actually being pumped. Although the blood flow dropped slightly, it confirmed that the light-induced cardiac muscle contraction rarely caused a pulse deficit in awake mice (Figure 5A).

We induced bradycardia (common in pacemaker candidates) in the mice using muscarine. With muscarine administration, the heart rate dropped to 150–300 BPM within 5 min (*n* = 3). The ECG in Figure 5B is a few minutes after administrating muscarine. By photostimulation, the heart rate increased to the photostimulation frequency (Figures 5B, C). There was a slight change in blood pressure during pacing, but there was no significant increase or decrease on average. (Figures 5B, C). As the drug effects lasted for a prolonged period, the heart rate returned after the photostimulation was stopped.

### Blood pressure depending on stimulation sites

To evaluate which heart regions were better for photostimulation, we photostimulated four different regions with a flashing rate of 600 f/m while measuring the blood pressure in anesthetized mice. Consequently, photostimulation of each region led to different ECG traces (Supplementary Figure S4B) and blood pressure (Supplementary Figure S4C). Atrial photostimulation triggered P waves that caused normal QRS waves. Ventricular photostimulation triggered wider QRS complexes, and P waves disappeared. Left ventricular photostimulation induced downward R waves since the current transmitted in the opposite direction from the normal/spontaneous heart contraction. By calculating the AUC per unit time in the blood pressure chart, we compared the amount of blood pressure change between baseline (immediately before each pacing) and during pacing. The AUC per unit time of baseline was set to 100%. (Supplementary Figure S4D). The baseline heart rates in each area before photostimulation were 555 ± 24.1 BPM in the right atrium (RA), 554 ± 22.9 BPM in the right ventricle (RV), 579 ± 25.4 BPM in the LV (upper), and 575 ± 24.6 BPM in the LV (lower).

## Discussion

In the present study, we established a cardiac photostimulation system in freely moving mice. We obtained detailed physiological information such as the respiration and blood pressure from mice undergoing optogenetic pacing for the first time. Furthermore, it allowed the contact-free termination of bradycardia through blue light illumination of the ventricular, not dependent on conventional electrostimulations or medicines. Thus, our physiological findings provide insights that can lead to the development of a new optogenetic-based cardiac pacing device. For further improvement of the results of this research, studies on wireless pacing technology and further device stabilization are required.

Using an optogenetic approach to the pacemaker has advantages in clinical application. First, we can contactlessly stimulate the cardiac muscle without pacing leads. Pacing leads attached to inside of the right atrium and the right ventricle in major conventional artificial pacemakers occasionally cause complications such as disconnection, cardiac perforation, or venous stenosis (Hirschl et al., 2007; Gul and Kayrak, 2011; Sadamatsu, 2011). Lead replacement surgery is required to address these complications, which has its own potential risks. Additionally, this surgery is difficult to perform in pediatric patients due to their small bodies (Gheissari et al., 1991) and must be needed as they grow. Therefore, electrical pacing with pacing leads is not optimal for situations that require longer treatment durations, such as infantile diseases. Second, we can stimulate specific cells. For these reasons, a leadless and non-electrical pacemaker may improve the quality of life of patients with pacemakers.

### AAV gene transfer

Previous studies have shown superior transgene expression mediated by AAV serotype 9 in some organs, including the heart (Zincarelli et al., 2008). Our study also showed that the transgene was highly expressed in the murine cardiac muscle using the AAV serotype 9. We were able to confirm long-term (>1 year) gene expression. For example, gene therapy drugs using AAV, such as Zolgensma^®^, are known to have a sustained, lifelong effect (Mendell et al., 2017). We selected the cTnT promoter that reported the gene expression is restricted to cardiomyocytes(Prasad et al., 2011) (Lin et al., 2015). Although previous studies have shown that the promoter used in this experiment induces cardiomyocyte-specific gene expression, it was also shown to be expressed in other non-cardiac muscles. Therefore, other myocardial-specific promoters also need to be explored.

### The effect of systemic AAV administration

AAVs were administered intraperitoneally to 3-day-old mice with an underdeveloped blood-peritoneal barrier. Intraperitoneal administration is simpler than intravenous administration or direct injection into the cardiac muscle, which require major surgeries such as thoracotomy, although intraperitoneal injection requires a larger volume. The newborn mice required smaller volumes than did the mature mice. Although, the most popular method of systemically delivering the genome is an intravenous injection, it requires a bunch of genome copies; 10^13∼14^ copies per mouse in adult mice (Xie et al., 2011). Consequently, intraperitoneal administration in newborn animals would likely be a less-invasive and an economical way to induce ubiquitous expression. During injection, we have to avoid the virus solution leaking from their small bodies. Leaking the solution makes the ChR2 expression rate would decrease.

### Device characterization

We developed a photostimulation device implantable in freely moving mice using a high power and minimal size LED chip. We found that the device’s surface temperature exceeded the normal body temperature at 900 f/m. However, this likely did not affect the mice because cardiac pacing is rarely performed for extended periods at a frequency beyond the baseline heart rate of 400–600 BPM. The surface temperature depends on conditions such as the property of the LED chips, components of the LED coating, pulse duration, and light intensity. Therefore, we should consider the effect of temperature each time these conditions are changed.

The novel LED device designed in the present study is connected to a pulse generator with a conductor. It would be more desirable to use a wireless device to not interfere with the mice’s normal behavior. This is one of the biggest technical problems pertaining to device attachment in our mouse model. Although attaching devices outside the thoracic cavity produces a minimally invasive implantation, the position gap between the device and the heart remains a problem. The device position is particularly important, because device misalignment leads to a displacement of the illumination site, resulting in pacing failure. The misalignment could be induced by changes in the posture of mice, thickening or adhesion of the connective tissue around the device, and/or loosening of the thread that holds the device in place.

### Conditions for optogenetic-based cardiac pacing

We successfully controlled the heart rate of freely moving mice using optogenetics. With sufficient expression levels of ChR2, we could perform cardiac pacing even with some light attenuation or misalignment. Several studies have shown that excitation starting from the photostimulation site spreads to the entire heart via gap junctions (Nussinovitch and Gepstein, 2015). We chose LV as the illumination site because it plays a significant role in pumping blood for the whole body.

Photostimulation at a frequency of 700–1,200 f/m was able to synchronize the heartbeats almost completely. The heart rate immediately returned to the baseline levels several milliseconds after stopping pulse illumination, indicating the reversibility of cardiac rhythm. A short pause in the ECG after photostimulation indicated a compensatory pause. The inability of the heart to respond to extremely high-frequency pulse illumination may be due to the refractory period of a cardiomyocyte.

The shapes of the ECG traces were different depending on the photostimulation site. This phenomenon was consistent with the electrical cardiac pacing and previous findings (Zaglia et al., 2015). The photostimulation site changed the blood pressure, and RA stimulation produced the best blood pressure. Because a cardiac spontaneous electrical excitation generally starts from the RA.

The heart rate cannot be reduced by illuminating an excitatory ion channel, ChR2, on cardiomyocytes. Other group reported that it could be reduced by illuminating an inhibitory ion pump, archaerhodopsin (Govorunova et al., 2016) or sympathetic nerves (Yu et al., 2017). Also illuminating ChR2 on the vagus nerves could reduce the heart rate (Chang et al., 2015; Rajendran et al., 2019).

### Effect on respiration

The respiratory rate did not directly correlate with the flashing rate. The Δ respiratory rate peaked at approximately 1,000 f/m and gradually decreased as the flashing rate increased from 1,000 f/m to approximately 1,400 f/m, and then further increased. This behavior seems to be similar to the capture rate (Figure 3D). For example, stimulating the heart at 1,400 f/m induced heartbeats once every two pulses resulted in approximately 700 BPM. In Figure 4C, the Δ respiratory rate at 700 f/m is close to it at 1,400 f/m. The respiratory rate is likely not affected by the flashing rate, but by the actual heart rate (Supplementary Figure S5, right-lower panel). It is known that breathing frequency is influenced by heart rate. Moreover, it is also known that a transient decrease in blood pressure can affect peripheral chemoreceptors and subsequently increase respiratory rate (McMullan, 2010). In our study, cardiac pacing led to a slight reduction in blood pressure. As described in the next section, the heart rate exceeding the baseline significantly tends to drop blood pressure. Therefore, the mechanical sensory from a heart stimulation or a perceived drop in blood pressure during pacing may have increased respiratory rate. To describe more accurate mechanism, it would be better to perform photostimulation with simultaneous blood pressure and respiration measurements.

### Blood pressure confirmation

We measured the blood pressure under pacing. Exceeding the physiological heart rate would result in inadequate blood supply to the ventricle. We confirmed that light-induced contraction of the cardiac muscle caused the blood to flow. It was difficult to measure blood pressure properly under poor conditions. Especially in experiments with bradycardia and site-specific pacing, their baseline blood pressure was low. Baseline blood pressure could be lowered due to hyposthenia caused by highly invasive surgery (thoracotomy) or by blood loss associated with the surgery (catheter insertion into the carotid artery). Too high a stimulation rate compared to the baseline heart rate induced lower blood pressure during photostimulation.

As mentioned above, blood pressure also differed depending on the illumination site. Illumination of the left ventricle reduced blood pressure lower than baseline. Even with electrical pacing, stimulation of the ventricle lowers blood pressure, especially with a high stimulation rate (Ouyang et al., 2019). Supplementary Figure S4, especially C, suggests that RA illumination is the least likely to reduce blood pressure, though there is no significant difference. Theoretically, the RA stimulation (or both the RA and RV) would be best. For technical reasons, stimulating the RA in freely moving mice was difficult since the RA was too small to sufficiently illuminate. Being able to photostimulate RA in freely moving mice is a challenge that needs to be addressed.

### Therapeutic effects on bradycardia and other arrhythmias

After inducing bradycardia in freely moving mice using drugs, photostimulation was applied at a frequency close to the baseline heart rate and increased heart rate without a pulse deficit. Previous studies have suggested that other arrhythmias, such as ventricular tachycardia and fibrillation, could also be treated using optogenetics. For example, continuously illuminating the heart with blue light for a few seconds (expressed excitatory opsin) has been shown to stop tachycardia (Bruegmann et al., 2016; Nyns et al., 2017; 2019). This function is similar to an implantable cardioverter-defibrillator. Additionally, photoinhibition of the sympathetic ganglia (expressed inhibitory opsin) prevented the animals from suffering a tachycardia attack (Yu et al., 2017). Most of these studies involved anesthetized animals. To evolve these devices, treatment must be achieved in freely moving mice.

### Electric vs. optogenetic-based cardiac pacing

The main advantage of optogenetic-based pacing is avoiding the surgery of pacing leads approaching inside the ventricle through blood vessels. This provides less invasive implantation and stimulation applied from outside the thoracic cavity. Furthermore, cell-type-specific stimulation could reduce the effects on other tissues. A disadvantage of optogenetic-based pacing is that it is difficult to keep the LED in position on the heart, as described above. There are also disadvantages related to ChR2: ChR2 is a non-selective cation channel. Therefore, it passes ions that affect a pH, such as hydrogen ions (Beppu et al., 2014). This may be solved by changing the opsin subtype. Problems associated with a pH occur in electrical stimulation as well as photostimulation. Merrill et al. found that gasses affecting the pH were generated by electrical stimulation (Merrill, Bikson, and Jefferys, 2005). In a previous study, no evidence of capture failure was found in long-term (2 h) studies with a continuous application of blue light (Nussinovitch and Gepstein, 2015). However, blue light has cytotoxicity. The possible risk of it could be reduced using red light-sensitive opsin. Red light additionally has a higher tissue permeability (Lin et al., 2013; Hsueh et al., 2023). Consequently, we suggest that optogenetic pacing may be superior to electrical pacing except the cytotoxicity.

## Limitations

Our device could do pacing manually, but it was not an automatic pacing system which is essential for artificial pacemakers. Also, it was difficult to achieve stable implantation for a long period (>1 month) with our implantation method. This was mainly due to the misalignment of the device. Therefore, there is room for further improvement of the illumination device structures and implantation methods.

We used mice as an animal model in our study because murine models support various pathological phenotypes, including cardiovascular diseases. Given that mice’s body structure differs from that of humans, further evaluations using larger animals are necessary. For example, device structures and implantation methods must be tailored according to animal species. From the perspective of a clinical setting, transduction of AAV to the cardiomyocytes also remains an issue.

## Data Availability

The original contributions presented in the study are included in the article/Supplementary Material, further inquiries can be directed to the corresponding author.

## References

[B1] Atasoy D. Aponte Y. Su H. H. Sternson S. M. (2008). A FLEX switch targets channelrhodopsin-2 to multiple cell types for imaging and long-range circuit mapping. J. Neurosci. 28 (28), 7025–7030. 10.1523/JNEUROSCI.1954-08.2008 18614669 PMC2593125

[B2] Bayliss C. E. Beanlands D. S. Baird R. J. (1968). The pacemaker-twiddler’s syndrome: A new complication of implantable transvenous pacemakers. Can. Med. Assoc. J. 99 (8), 371–373. Available at: http://www.ncbi.nlm.nih.gov/pubmed/4952398 (Accessed June 25, 2021). 4952398 PMC1924435

[B3] Beppu K. Sasaki T. Tanaka K. F. Yamanaka A. Fukazawa Y. Shigemoto R. (2014). Optogenetic countering of glial acidosis suppresses glial glutamate release and ischemic brain damage. Neuron 81 (2), 314–320. 10.1016/j.neuron.2013.11.011 24462096

[B4] Boyden E. S. Zhang F. Bamberg E. Nagel G. Deisseroth K. (2005). Millisecond-timescale, genetically targeted optical control of neural activity. Nat. Neurosci. 8 (9), 1263–1268. 10.1038/nn1525 16116447

[B5] Boyle P. M. Karathanos T. V. Trayanova N. A. (2018). Cardiac optogenetics 2018. JACC Clin. Electrophysiol. 4, 155–167. 10.1016/j.jacep.2017.12.006 29749932 PMC5951179

[B6] Bruegmann T. Boyle P. M. Vogt C. C. Karathanos T. V. Arevalo H. J. Fleischmann B. K. (2016). Optogenetic defibrillation terminates ventricular arrhythmia in mouse hearts and human simulations. J. Clin. Investigation 126 (10), 3894–3904. 10.1172/JCI88950 PMC5096832 27617859

[B7] Bruegmann T. Malan D. Hesse M. Beiert T. Fuegemann C. J. Fleischmann B. K. (2010). Optogenetic control of heart muscle *in vitro* and *in vivo* . Nat. Methods 7 (11), 897–900. 10.1038/nmeth.1512 20881965

[B8] Bruegmann T. Van Bremen T. Vogt C. C. Send T. Fleischmann B. K. Sasse P. (2015). Optogenetic control of contractile function in skeletal muscle. Nat. Commun. 6, 7153. 10.1038/ncomms8153 26035411 PMC4475236

[B9] Chang R. B. Strochlic D. E. Williams E. K. Umans B. D. Liberles S. D. (2015). Vagal sensory neuron subtypes that differentially control breathing. Cell 161 (3), 622–633. 10.1016/j.cell.2015.03.022 25892222 PMC4842319

[B10] Dalex M. Malezieux A. Parent T. Zekry D. Serratrice C. (2021). Phrenic nerve stimulation, a rare complication of pacemaker: A case report. Medicine 100 (11), e25060. 10.1097/MD.0000000000025060 33725981 PMC7982205

[B11] Gheissari A. Hordof A. J. Spotnitz H. M. (1991). Transvenous pacemakers in children: Relation of lead length to anticipated growth. Ann. Thorac. Surg. 52 (1), 118–121. 10.1016/0003-4975(91)91431-T 2069438

[B12] Govorunova E. G. Cunha S. R. Sineshchekov O. A. Spudich J. L. (2016). Anion channelrhodopsins for inhibitory cardiac optogenetics. Sci. Rep. 6, 33530. 10.1038/srep33530 27628215 PMC5024162

[B13] Gul E. E. Kayrak M. (2011). “Common pacemaker problems: Lead and pocket complications,” in Modern pacemakers (London, United Kingdom: Intechopen). 10.5772/556

[B14] Gutruf P. Yin R. T. Lee K. B. Ausra J. Brennan J. A. Qiao Y. (2019). Wireless, battery-free, fully implantable multimodal and multisite pacemakers for applications in small animal models. Nat. Commun. 10 (1), 5742. 10.1038/s41467-019-13637-w 31848334 PMC6917818

[B15] Hirschl D. A. Jain V. R. Spindola-Franco H. Gross J. N. Haramati L. B. (2007). Prevalence and characterization of asymptomatic pacemaker and ICD lead perforation on CT. PACE - Pacing Clin. Electrophysiol. 30 (1), 28–32. 10.1111/j.1540-8159.2007.00575.x 17241311

[B16] Hsueh B. Chen R. Jo Y. Tang D. Raffiee M. Kim Y. S. (2023). Cardiogenic control of affective behavioural state. Nature 615, 292–299. 10.1038/s41586-023-05748-8 36859543 PMC9995271

[B17] Ikoma Y. Kusumoto-Yoshida I. Yamanaka A. Ootsuka Y. Kuwaki T. (2018). Inactivation of serotonergic neurons in the rostral medullary raphé attenuates stress-induced tachypnea and tachycardia in mice. Front. Physiology 9, 832. 10.3389/fphys.2018.00832 PMC6050454 30050449

[B18] Li J. Wang L. Luo J. Li H. Rao P. Cheng Y. (2021). Optical capture and defibrillation in rats with monocrotaline-induced myocardial fibrosis 1 year after a single intravenous injection of adeno-associated virus channelrhodopsin-2. Heart rhythm. 18 (1), 109–117. 10.1016/j.hrthm.2020.08.002 32781160

[B19] Li X. Gutierrez D. V. Hanson M. G. Han J. Mark M. D. Chiel H. (2005). Fast noninvasive activation and inhibition of neural and network activity by vertebrate rhodopsin and green algae channelrhodopsin. Proc. Natl. Acad. Sci. U. S. A. 102 (49), 17816–17821. 10.1073/pnas.0509030102 16306259 PMC1292990

[B20] Lin J. Y. Knutsen P. M. Muller A. Kleinfeld D. Tsien R. Y. (2013). ReaChR: A red-shifted variant of channelrhodopsin enables deep transcranial optogenetic excitation. Nat. Neurosci. 16 (10), 1499–1508. 10.1038/nn.3502 23995068 PMC3793847

[B21] Lin Z. Zhou P. Von Gise A. Gu F. Ma Q. Chen J. (2015). Pi3kcb links Hippo-YAP and PI3K-AKT signaling pathways to promote cardiomyocyte proliferation and survival. Circulation Res. 116 (1), 35–45. 10.1161/CIRCRESAHA.115.304457 25249570 PMC4282610

[B22] McMullan S. Pilowsky P. M. (2010). The effects of baroreceptor stimulation on central respiratory drive: A review. Respir. Physiology Neurobiol. 174 (1–2), 37–42. 10.1016/J.RESP.2010.07.009 20674807

[B23] Mendell J. R. Al-Zaidy S. Shell R. Arnold W. D. Rodino-Klapac L. R. Prior T. W. (2017). Single-dose gene-replacement therapy for spinal muscular atrophy. N. Engl. J. Med. 377 (18), 1713–1722. 10.1056/nejmoa1706198 29091557

[B24] Merrill D. R. Bikson M. Jefferys J. G. R. (2005). Electrical stimulation of excitable tissue: Design of efficacious and safe protocols. J. Neurosci. Methods 141, 171–198. 10.1016/j.jneumeth.2004.10.020 15661300

[B25] Nagel G. Brauner M. Liewald J. F. Adeishvili N. Bamberg E. Gottschalk A. (2005). Light activation of Channelrhodopsin-2 in excitable cells of caenorhabditis elegans triggers rapid behavioral responses. Curr. Biol. 15 (24), 2279–2284. 10.1016/j.cub.2005.11.032 16360690

[B26] Nagel G. Szellas T. Huhn W. Kateriya S. Adeishvili N. Berthold P. (2003). Channelrhodopsin-2, a directly light-gated cation-selective membrane channel. Proc. Natl. Acad. Sci. U. S. A. 100 (2), 13940–13945. 10.1073/pnas.1936192100 14615590 PMC283525

[B27] Nussinovitch U. Gepstein L. (2015). Optogenetics for *in vivo* cardiac pacing and resynchronization therapies. Nat. Biotechnol. 33 (7), 750–754. 10.1038/nbt.3268 26098449

[B28] Nyns E. C. A. Kip A. Bart C. I. Plomp J. J. Zeppenfeld K. Schalij M. J. (2017). Optogenetic termination of ventricular arrhythmias in the whole heart: Towards biological cardiac rhythm management. Eur. Heart J. 38 (27), 2132–2136. 10.1093/eurheartj/ehw574 28011703 PMC5837774

[B29] Nyns E. C. A. Poelma R. H. Volkers L. Plomp J. J. Bart C. I. Kip A. M. (2019). An automated hybrid bioelectronic system for autogenous restoration of sinus rhythm in atrial fibrillation. Sci. transrational Med. 11, eaau6447. 10.1126/scitranslmed.aau6447 30814339

[B30] Ouyang H. Liu Z. Li N. Shi B. Zou Y. Xie F. (2019). Symbiotic cardiac pacemaker. Nat. Commun. 10 (1), 1821. 10.1038/s41467-019-09851-1 31015519 PMC6478903

[B31] Prasad K. M. M. R. Xu Y. Yang Z. Acton S. T. French B. A. (2011). Robust cardiomyocyte-specific gene expression following systemic injection of AAV: *In vivo* gene delivery follows a Poisson distribution. Gene Ther. 18 (1), 43–52. 10.1038/gt.2010.105 20703310 PMC2988989

[B32] Rajendran P. S. Challis R. C. Fowlkes C. C. Hanna P. Tompkins J. D. Jordan M. C. (2019). Identification of peripheral neural circuits that regulate heart rate using optogenetic and viral vector strategies. Nat. Commun. 10 (1), 1944. 10.1038/s41467-019-09770-1 31028266 PMC6486614

[B33] Sadamatsu K. (2011). “Complication of pacemaker implantation: An atrial lead perforation,” in Modern pacemakers - present and future (London, United Kingdom: Intechopen), 333–342. 10.5772/13854

[B34] Tanabe K. Kotoda M. Nakashige D. Mitsui K. Ikemoto K. Matsukawa T. (2019). Sudden onset pacemaker-induced diaphragmatic twitching during general anesthesia. JA Clin. Rep. 5 (1), 36. 10.1186/s40981-019-0257-7 32026968 PMC6967311

[B35] Vogt C. C. Bruegmann T. Malan D. Ottersbach A. Roell W. Fleischmann B. K. (2015). Systemic gene transfer enables optogenetic pacing of mouse hearts. Cardiovasc. Res. 106 (2), 338–343. 10.1093/cvr/cvv004 25587047

[B36] Xie J. Xie Q. Zhang H. Ameres S. L. Hung J.-H. Su Q. (2011). MicroRNA-regulated, systemically delivered rAAV9: A step closer to CNS-restricted transgene expression. Mol. Ther. J. Am. Soc. Gene Ther. 19 (3), 526–535. 10.1038/mt.2010.279 PMC3048189 21179009

[B37] Yu L. Zhou L. Cao G. Po S. S. Huang B. Zhou X. (2017). Optogenetic modulation of cardiac sympathetic nerve activity to prevent ventricular arrhythmias. J. Am. Coll. Cardiol. 70 (22), 2778–2790. 10.1016/j.jacc.2017.09.1107 29191327

[B38] Zaglia T. Pianca N. Borile G. Da Broi F. Richter C. Campione M. (2015). Optogenetic determination of the myocardial requirements for extrasystoles by cell type-specific targeting of ChannelRhodopsin-2. Proc. Natl. Acad. Sci. U. S. A. 112 (32), E4495–E4504. 10.1073/pnas.1509380112 26204914 PMC4538656

[B39] Zhang W. Sakurai T. Fukuda Y. Kuwaki T. (2006). Orexin neuron-mediated skeletal muscle vasodilation and shift of baroreflex during defense response in mice. Am. J. Physiol. Regul. Integr. Comp. Physiol. 290, 1654–1663. 10.1152/ajpregu.00704.2005 16410401

[B40] Zincarelli C. Soltys S. Rengo G. Rabinowitz J. E. (2008). Analysis of AAV serotypes 1-9 mediated gene expression and tropism in mice after systemic injection. Mol. Ther. 16 (6), 1073–1080. 10.1038/mt.2008.76 18414476

