# Supplementary figures and images for: The physiological response during optogenetic-based cardiac pacing in awake freely moving mice
Source: Front Physiol. 2023 Sep 6;14:1130956. doi: 10.3389/fphys.2023.1130956 (PMC10509767; doi:10.3389/fphys.2023.1130956)

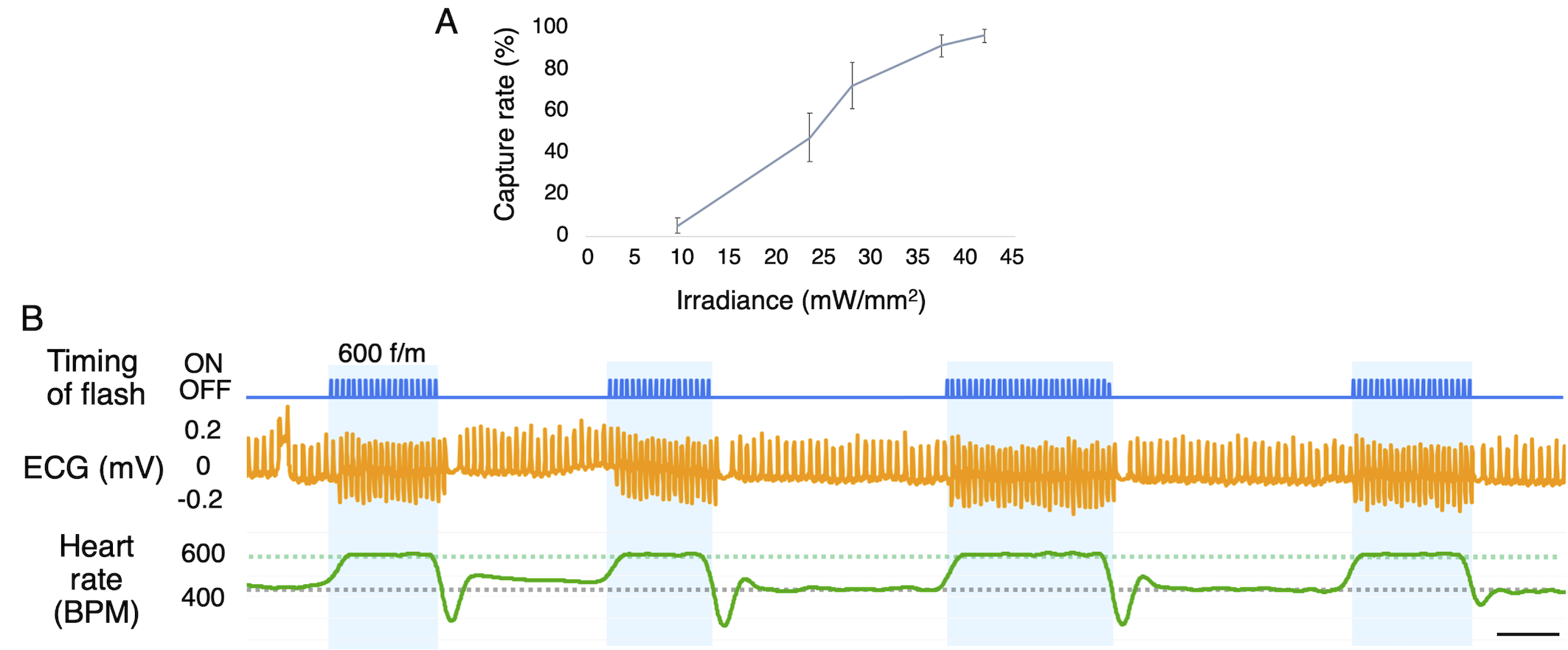

Supplement: Supplementary file 1 [file Image3.TIFF]

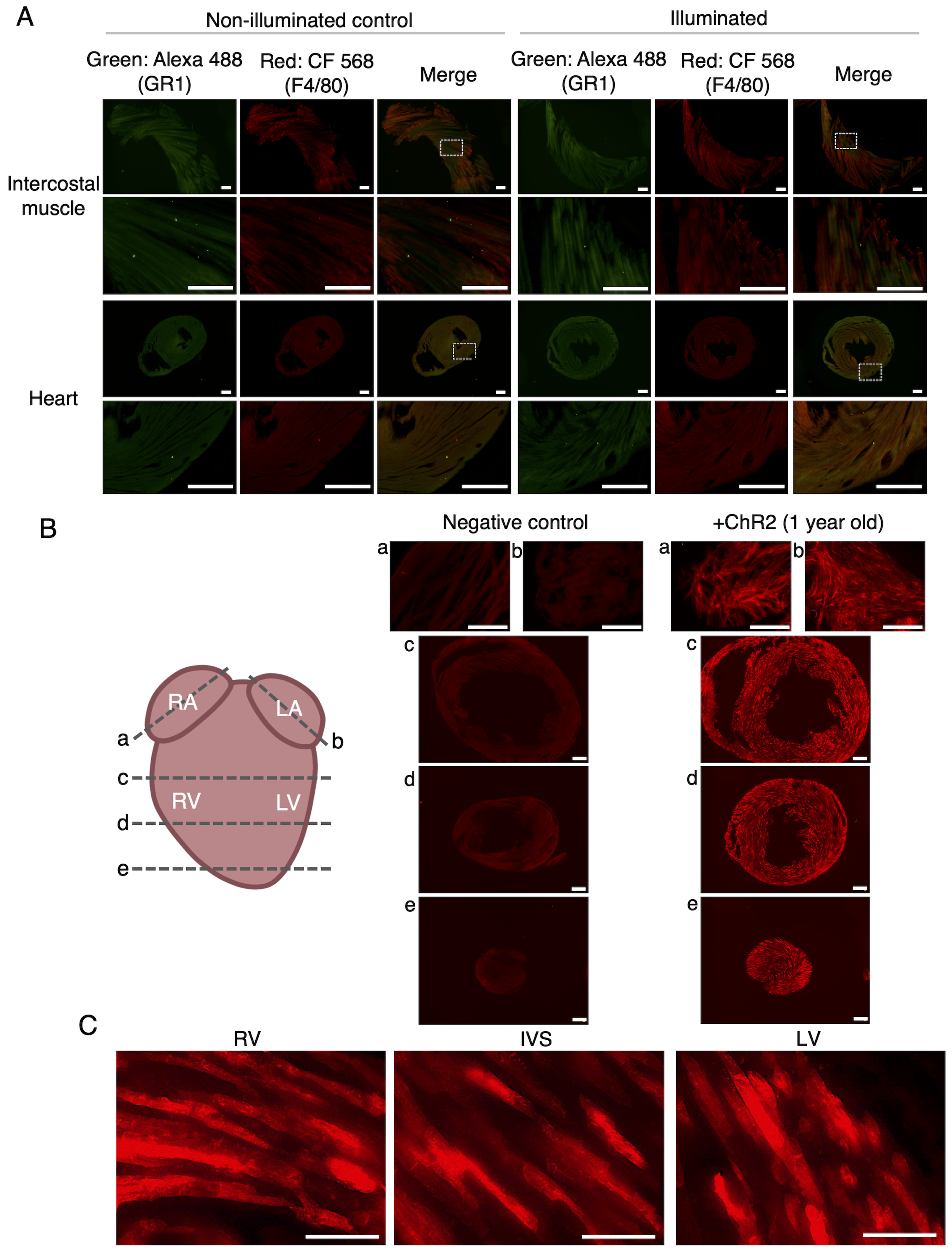

Supplement: Supplementary file 2 [file Image1.TIFF]

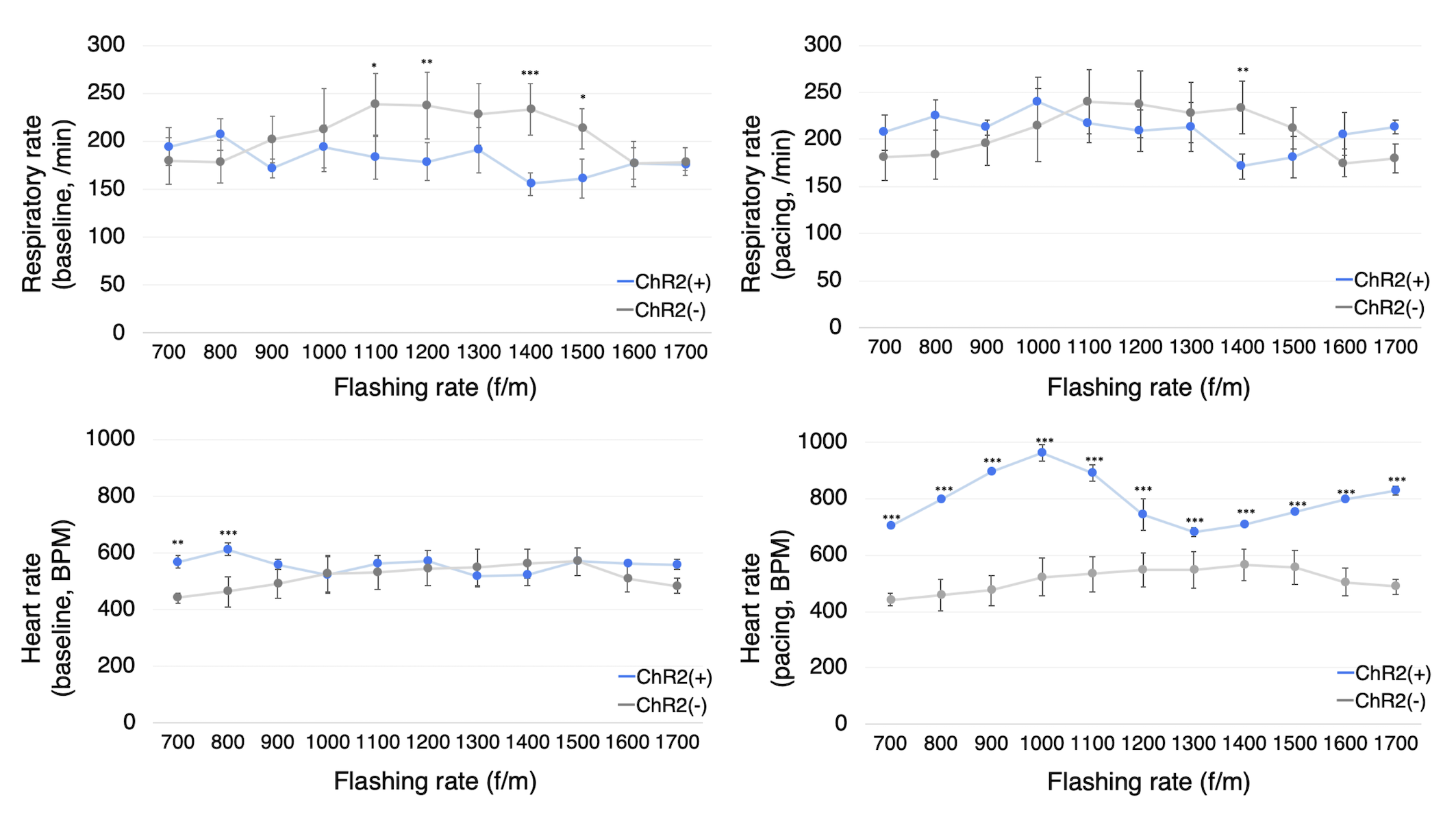

Supplement: Supplementary file 5 [file Image5.TIFF]

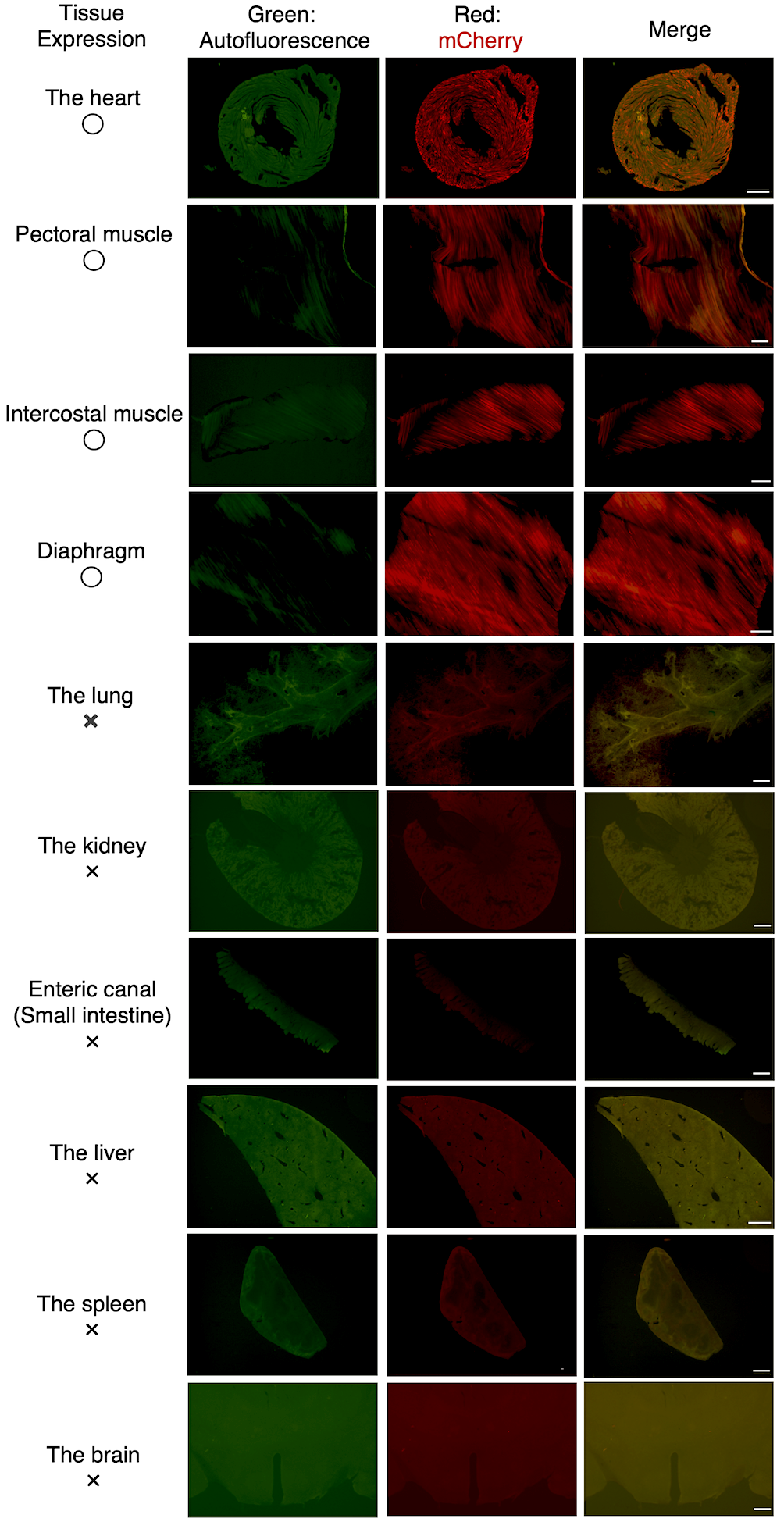

Supplement: Supplementary file 6 [file Image2.TIFF]

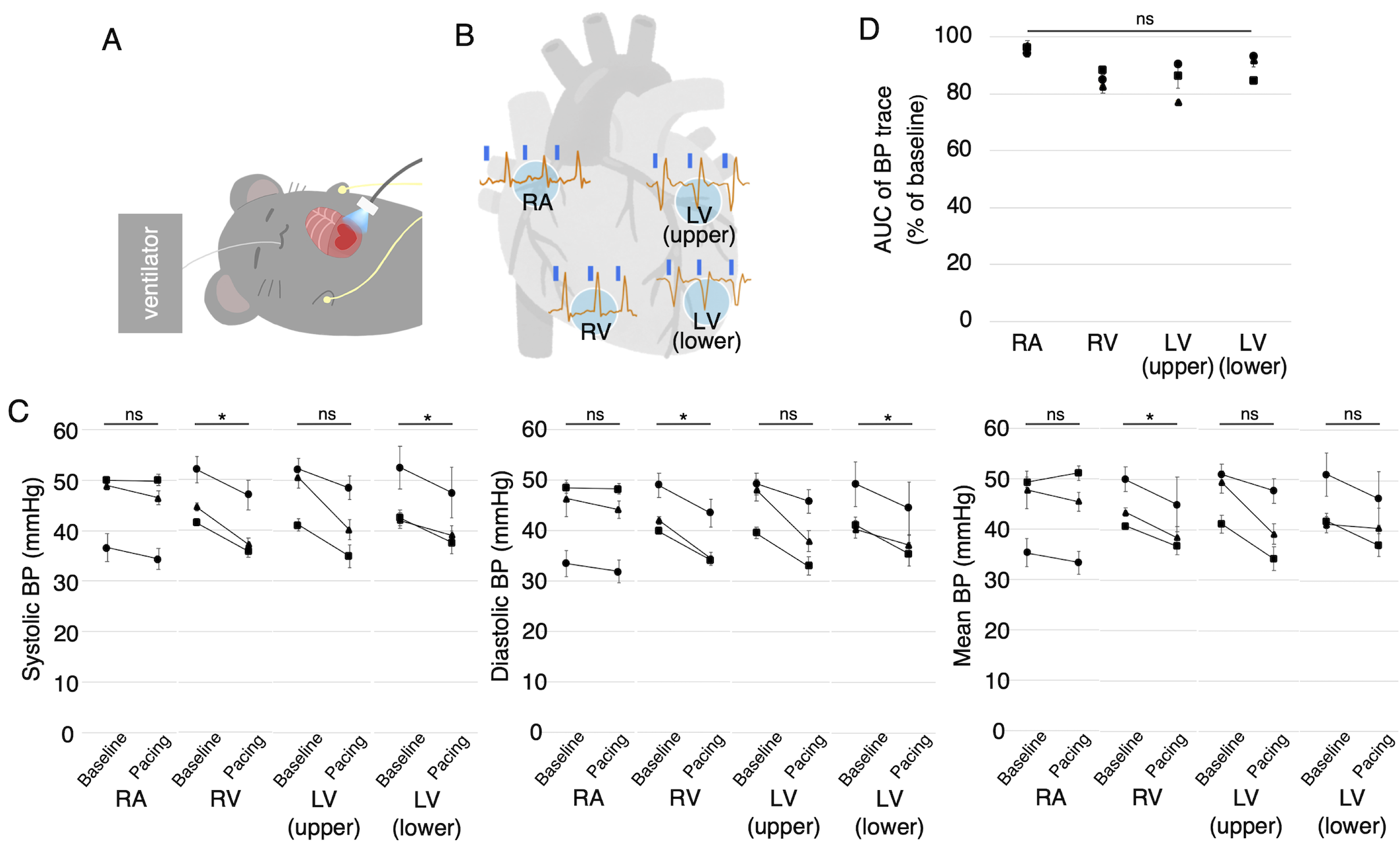

Supplement: Supplementary file 7 [file Image4.TIFF]
