# Supplementary material for: The physiological response during optogenetic-based cardiac pacing in awake freely moving mice
Source: Front Physiol. 2023 Sep 6;14:1130956. doi: 10.3389/fphys.2023.1130956 (PMC10509767; doi:10.3389/fphys.2023.1130956)
Supplement: Supplementary file 4 [file Table1.DOCX]

**Supplementary Figure Legends**

**Supplementary Figure 1. Details of gene expression.**

**(A)** Histological experiment to evaluate inflammation in the intercostal muscle and heart following 30 min of LED illumination at 900 f/m (right) compared to non-illuminated control (left). Tissue sections were collected two days after LED illumination. The magnified images in the bottom row of each panel represent the area indicated by the square in the top row. Green: GR1 (a neutrophil marker), Red: F4/80 (a macrophage marker). Scale bar: 500 μm. **(B)** Representative section images of the heart at five locations. The left image shows the five locations. The right images compare negative control and ChR2-expressed slice samples. Red: mCherry. Scale bar: 500 μm. **(C)** Higher magnification images of three parts of the ventricle. Scale bar: 100 μm.

RA, right atrium; LA, left atrium; RV, right ventricle; LV, left ventricle; IVS, interventricular septum.

**Supplementary Figure 2.** Fluorescence images of several organ samples eight weeks after systemic administration of adeno-associated viruses (AAVs). Slice samples obtained after systemic administration of the AAVs encoding the cTnT promoter (AAV-cTnT-Cre and AAV-CAG-FLEX-ChR2-mCherry). Expression in the cardiac and skeletal muscles was noted. Scale bar: 500 μm.

**Supplementary figure 3**

**(A)** Dose-response curve summarizing the correlation between LED irradiance and capture rate. Data are presented as the mean ± SEM (total 12 trials in 3 mice). **(B)** Representative example of repetitive pacing. All ChR2-expressed mice used in this study could be photostimulated repeatedly. Scale bar: 1 sec.

**Supplementary Figure 4. Site-specific optical pacing during blood pressure measurement.**

**(A)** Schematic illustration shows how to photostimulate. Only site-specific optical pacing was performed under thoracotomy.

**(B)** ECG traces of originated by light stimulation of different regions of the myocardium. **(C)** Changes in blood pressure compared to baseline (before pacing). Each dot represents the average of 2-11 samples from one mouse (n=3). Data are presented as the mean ± SEM (*p < 0.05, paired *t*-test). **(D)** Percentage of changes in AUC of blood pressure trace per unit time compared to baseline (before pacing). Each dot represents the average of 2-11 samples from one mouse (n=3). Data are presented as the mean ± SEM. Repeated measures ANOVA indicated there was no significant statistical difference among regions. Tukey’s multiple comparison test was used as a post hoc test.

RA, right atrium; RV, right ventricle; LV, left ventricle; BP, blood pressure; SEM, standard error of the mean; ANOVA, analysis of variance.

**Supplementary Figure 5.** Comparison of the physiological status of ChR2(+) (n = 7) and ChR2(-) (n = 4) animals at baseline and during illumination. The top row is a comparison of respiratory rate, and the bottom row is a comparison of heart rate. The left panels show the baseline comparison, and the right panels show the comparison during illumination. Data were collected after the heart rate stabilized. Data are presented as the mean ± SEM. Two-way ANOVA indicated that there was a significant statistical difference among animals (*p < 0.05, **p < 0.01, ***p < 0.001).

**Supplementary Movie.** A mouse undergoing photostimulation. Red line: ECG, blue line: timing of flash, green line: heart rate (BPM).
